# Presumed *Candida tropicalis* endogenous endophthalmitis with multifocal systemic *Candida albicans* abscesses: a case report

**DOI:** 10.3389/fmed.2026.1951092

**Published:** 2026-09-16

**Authors:** Tian Jingjing, Ye Nannan, Zhai Jingjie, Cui Ping, Zhang Yankun

**Affiliations:** Department of Ophthalmology, Hebei Chest Hospital, Shijiazhuang, China

**Keywords:** *Candida albicans*, *Candida tropicalis*, endogenous fungal endophthalmitis, metagenomic next-generation sequencing, vitrectomy

## Abstract

**Background:**

Endophthalmitis is a kind of infectious diseases that seriously threatens vision; Endogenous fungal endophthalmitis (EFE) accounts for 2% to 8% of all endophthalmitis, and is rare and often misdiagnosed in clinical practice. Different strains of *Candida* may have organ orientation differences, and mixed infections have also been reported in literature.

**Case report:**

A 72 year old male patient was admitted to the hospital due to fever for 5 months and decreased vision in both eyes. The patient's left vitreous fluid underwent metagenomic next-generation sequencing (mNGS), which revealed the presence of *Candida tropicalis* (1 sequence detected); The fungal culture of vitreous humor and the blood culture sent for testing at the same time was negative. *Candida albicans* was detected in the culture of surgical specimens for renal and lumbar lesions. The core clinical feature of this case is the inconsistency of pathogen detection between the whole body and the eyes. Due to mNGS only detected one *Candida tropicalis* sequence and the vitreous fungal culture was negative, this case should be considered as a suspected *Candida tropicalis* endophthalmitis clinically diagnosed. It may be a mixed invasive infection of *Candida albicans* and *Candida tropicalis*, and there may be differences in organ orientation between the two strains (*Candida albicans* prefers kidney and bone tissues, while *Candida tropicalis* is invasive to intraocular tissues), forming a zoning colonization pattern. However, this explanation still lacks conclusive microbiological evidence. The patient received local and systemic antifungal treatment in both eyes, and underwent bilateral vitrectomy. The systemic infection was controlled, and the left eye vision improved from near blindness to 0.25 (8 months) with some recovery in visual function.

**Conclusion:**

EFE is prone to misdiagnosis, and high-risk individuals should undergo pathogen testing as soon as possible. mNGS is a rapid identification tool, but caution should be exercised when single sequence detection and negative culture occur. When the pathogens are inconsistent, alternative explanations such as contamination and sequential infection should be excluded. The combination of comprehensive antifungal therapy and surgical treatment is effective,Clinical physicians need to establish a global mindset and pay attention to the intrinsic relationship between systemic lesions and eye damage.

## 1 Introduction

The core pathogenesis of EFE involves hematogenous spread of fungi from a systemic focus of infection to the eye, where they colonize and give rise to intraocular infection (1, 2). It is therefore a hematogenous infectious disease. Early diagnosis is challenging, and the reported misdiagnosis rate is as high as 16%–50% (3). Before the onset of ocular disease, the patient had undergone surgery for renal calculi and renal abscess. A large amount of purulent material was identified in the ureter, and culture of the submitted specimen yielded *Candida albicans*. The local physicians regarded this as colonization and did not initiate antifungal therapy. Soon thereafter, the patient developed low back pain and blurred vision. Cultures of specimens obtained during subsequent lumbar spine surgery also grew *Candida albicans* (++), suggesting the presence of multiple foci of infection. Fungi then enter the eye through systemic circulation. The metagenomic next-generation sequencing (mNGS) of the vitreous body ultimately considers the diagnosis of bilateral endogenous fungal endophthalmitis suspected to be caused by *Candida tropicalis*. The case is reported as follows.

## 2 Clinical data

### 2.1 General information

The patient was a 72-year-old man who frequently worked in high-temperature environments. He had a history of hypertension and diabetes mellitus; both blood pressure and blood glucose had been acceptably controlled with regular medication. He presented to our hospital in November 2025 with intermittent fever for 5 months, blurred vision in the right eye for more than 4 months, and blurred vision in the left eye for 1 month. Five months earlier, the patient began to experience recurrent fever, with a peak temperature of 38.7 °C. Symptomatic treatment with anti-inflammatory and antipyretic agents at a local clinic failed to provide improvement. He subsequently presented to the People's Hospital of Xingtai, where he was diagnosed with ‘renal calculi’ and ‘renal abscess' and underwent surgical treatment; a large amount of purulent material was noted intraoperatively. One week after surgery, the patient developed bilateral low back pain that progressively worsened, and he was transferred to Xingtai Mining Bureau Hospital. Tuberculous infection was suspected, but there was still no improvement after antituberculous therapy. Four months earlier, the patient developed blurred vision in the right eye and presented to Hebei Eye Hospital, where he was diagnosed with ‘uveitis of the right eye.’ Symptomatic treatment failed to relieve the blurred vision, which gradually worsened. One month earlier, blurred vision developed in the left eye, while the low back pain and fever progressively intensified. He then presented to the Department of Orthopedics at our hospital, where he was diagnosed with ‘lumbar abscess' and treated with systemic anti-inflammatory therapy. During hospitalization, the blurred vision in both eyes further deteriorated, and an ophthalmology consultation was requested. Ophthalmic examination showed the following: visual acuity was no light perception (NLP) in the right eye and hand motion (HM) close to face in the left eye; intraocular pressure was 9 mmHg in the right eye and 10 mmHg in the left eye. Anterior segment examination revealed no conjunctival hyperemia in the right eye; the cornea was clear; aqueous flare was present (+), with a small number of floating cells; the pupil measured approximately 2 mm in diameter; there were circumferential posterior synechiae, depigmentation at the pupillary margin, and lens opacity; In the left eye, fibrovascular proliferation of the nasal bulbar conjunctiva extended approximately 2 mm across the limbus onto the cornea; mixed conjunctival hyperemia was graded as (++); dust-like keratic precipitates (KPs) were visible on the inferior corneal endothelium; aqueous flare was (+++), with a large number of floating cells; the pupil measured approximately 2 mm in diameter; there were circumferential posterior synechiae, depigmentation at the pupillary margin, and lens opacity (Figures 1A–C). Fundus examination showed dense vitreous opacities in both eyes, with clumped white vitreous opacities visible within the vitreous cavity, and no clear fundus view. The diagnoses were as follows: 1. bilateral uveitis; 2. exudative retinal detachment in the right eye? 3. choroidal detachment in the right eye; 4. bilateral cataract. On the same day, local ocular anti-inflammatory and mydriatic therapy was initiated with Tobramycin and Dexamethasone Eye Drops, Diclofenac Sodium Ophthalmic Solution, and Compound Tropicamide Eye Drops. Meanwhile, an urgent vitreous tap of the left eye was performed, and vitreous fluid was obtained for metagenomic next-generation sequencing (mNGS) and cytokine testing.

**Figure 1:**
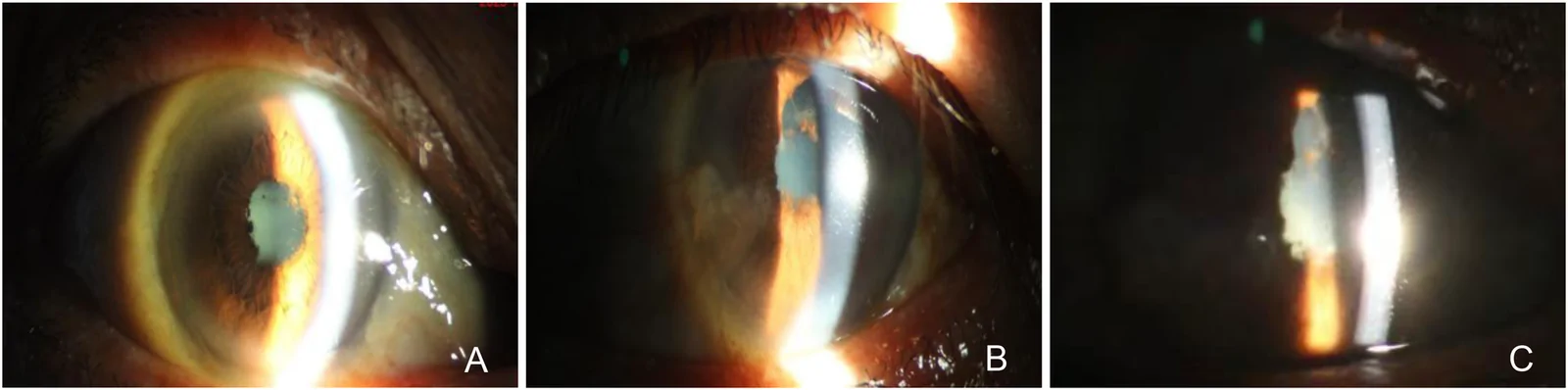
Photography of the anterior segment during initial diagnosis: right eye aqueous humor flashes (+), a small amount of floating matter, pupil diameter of about 2 mm, iris adhesions all around, visible depigmentation at the pupil edge, lens opacity **(A)**, mixed congestion of the left eye conjunctiva (++), transparent cornea, visible dust like corneal deposition (KP) below, normal anterior chamber depth, aqueous humor flashes (++++), a large amount of floating matter, drug-induced dilation of the pupil, iris adhesions at the pupil edge, visible depigmentation at the pupil edge, lens opacity **(B)**, enlarged left eye details show aqueous humor flashes (+++), and a large amount of floating matter **(C).**

### 2.2 Imaging and laboratory examinations

Ocular B-scan ultrasonography revealed vitreous opacities in both eyes and possible retinal detachment in the right eye? (Figures 2A,B). One day after submission of the specimen, vitreous fluid cytokine testing showed markedly elevated levels of interleukin-6 (IL-6), interleukin-8 (IL-8), and interleukin-10 (IL-10) (Table 1). Two days after specimen submission, mNGS of the vitreous fluid identified a suspected pathogen: *Candida genus* (series number 3), *Candida tropicalis* (sequence read count, 1) (Figure 3). Routine blood testing showed an elevated white blood cell count of 11.2 × 10^9^/L and an increased neutrophil percentage of 78.5%; liver and renal function, electrolytes, coagulation function, and related parameters were essentially within normal limits. In this case, the vitreous humor of the patient exhibited extremely elevated levels of IL-6 (4,962.94 pg/mL) and IL-8 (1,854.56 pg/mL), with a mild increase in IL-10 (26.37 pg/mL) and an IL-10/IL-6 ratio of only about 0.005. Studies have confirmed that significantly elevated levels of IL-6 and IL-8 are important biomarkers for infectious endophthalmitis, and fungal infections can induce their high expression; a low IL-10/IL-6 ratio can effectively exclude primary vitreoretinal lymphoma (usually >1.0) (4, 5), supporting an infectious diagnosis. This cytokine profile, coupled with the detection of *Candida tropicalis* by mNGS, mutually corroborates the Clinical diagnosis of acute severe fungal endophthalmitis. The levels of pro-inflammatory factors are highly consistent with the patient's clinical manifestations of severe vitreous opacity and visual acuity reduced to hand movement/no light perception in both eyes.

**Figure 2:**
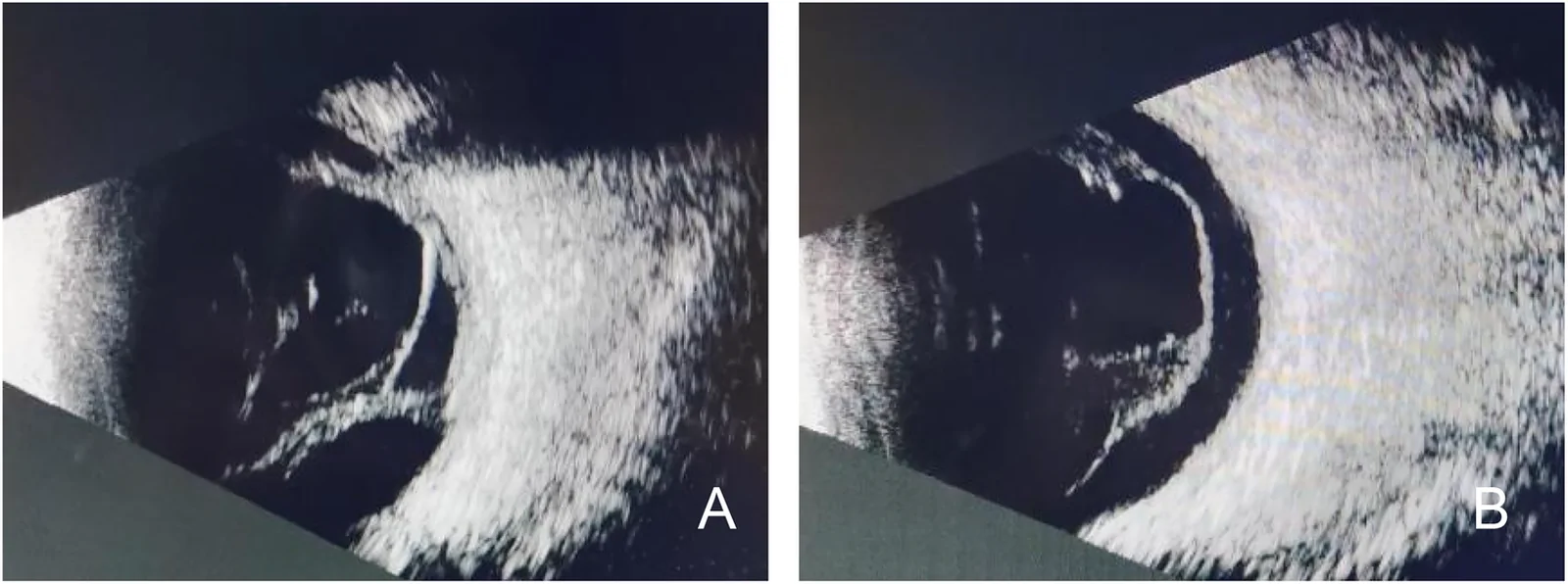
Bilateral ultrasound: right eye vitreous opacity, right eye retinal detachment? **(A),**left eye vitreous opacity **(B).**

**Table 1:** Vitreous fluid cytokine testing results.

Test item	Test result	Unit	Reference range
IL-6	4,962.94	pg/mL	≤5.4
IL-8	1,854.56	pg/mL	≤20.6
IL-10	26.37	pg/mL	≤12.9
IL-10/6	0.01		＜1.0

**Figure 3:**
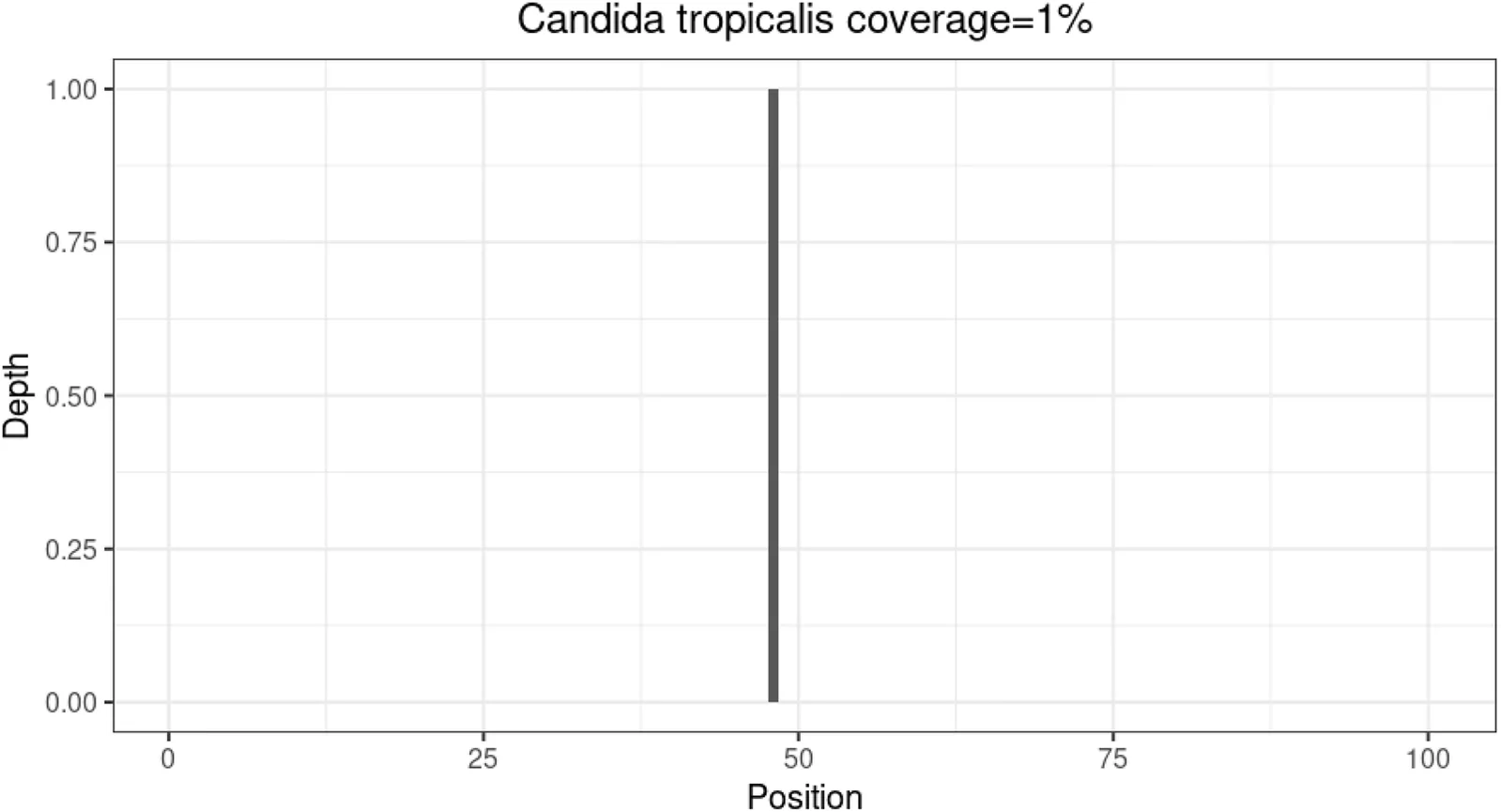
Pathogenic metagenomic sequencing detection of tropical *Candida:* the horizontal axis represents the average division of the microbial reference genome sequence into 100 intervals, and the vertical axis represents the number of sequences detected in different genome intervals.

### 2.3 Clinical diagnosis and treatment

Based on the patient's medical history, ophthalmological examination results, and metagenomic testing results of vitreous fluid, a diagnosis of endogenous fungal endophthalmitis in both eyes was considered. Because the patient had recurrent fever and was in poor general condition at that time, with a relatively severe lumbar abscess requiring priority lumbar surgery, bilateral ocular surgery could not be performed in a timely manner. He was therefore treated with bilateral intravitreal voriconazole injections (0.1 mg/0.1 mL) twice weekly, together with oral voriconazole tablets at a loading dose of 400 mg every 12 h followed by a maintenance dose of 200 mg every 12 h, while topical anti-inflammatory and mydriatic therapy was continued. One week later, the patient underwent surgical treatment for the lumbar abscess. Culture of the intraoperative specimen yielded *Candida albican*s ++ after 7 days. Because the ocular condition progressively worsened, the patient was transferred to the Department of Ophthalmology for further management. As *Candida albicans* had been identified at two systemic sites, namely the kidney and lumbar spine, while *Candida tropicalis* was detected in the eyes, the patient was considered to have an invasive fungal infection with systemic involvement. Systemic antifungal therapy was therefore intensified with intravenous caspofungin, administered as a single 70 mg loading dose on day 1 followed by 50 mg once daily thereafter(Stop taking medication in two weeks). Pars plana vitrectomy, lensectomy, and silicone oil tamponade were then performed in both eyes. Intraoperatively, diffuse, dense clumped purulent emboli were observed within the vitreous cavity of both eyes; these were thick in consistency, tightly adherent to the retinal surface, and formed white membranous changes (Figure 4A). After thorough removal, fundus examination revealed scattered retinal breaks with total retinal detachment in the right eye, whereas in the left eye a white mass-like lesion was seen temporal to the macula, arising from the subretinal space (red arrow), breaching the retina, and extending into the preretinal space (Figures 4B,C). Intravitreal voriconazole injection was administered postoperatively. Because the patient had fungal infection at multiple systemic sites, systemic antifungal therapy was required for a full 12-week course. At the 3-month postoperative follow-up, the patient's condition remained stable, with no recurrence. Discontinue the use of voriconazole. After silicone oil removal and intraocular lens implantation in the left eye, visual acuity improved to 0.2, with a favorable outcome and high patient satisfaction. Due to the right eye still lacking light perception, the intraocular pressure is 10mmHg. The retinal reattachment is inadequate, and it is in a silicone oil-filled state. The ocular condition is stable, with no signs of infection. Considering the patient's right eye has no vision, the silicone oil removal surgery will not be performed temporarily. On re-examination 3 months later, the white mass-like lesion temporal to the macula in the left eye had regressed markedly compared with the previous examination (Figures 5A,B).

**Figure 4:**
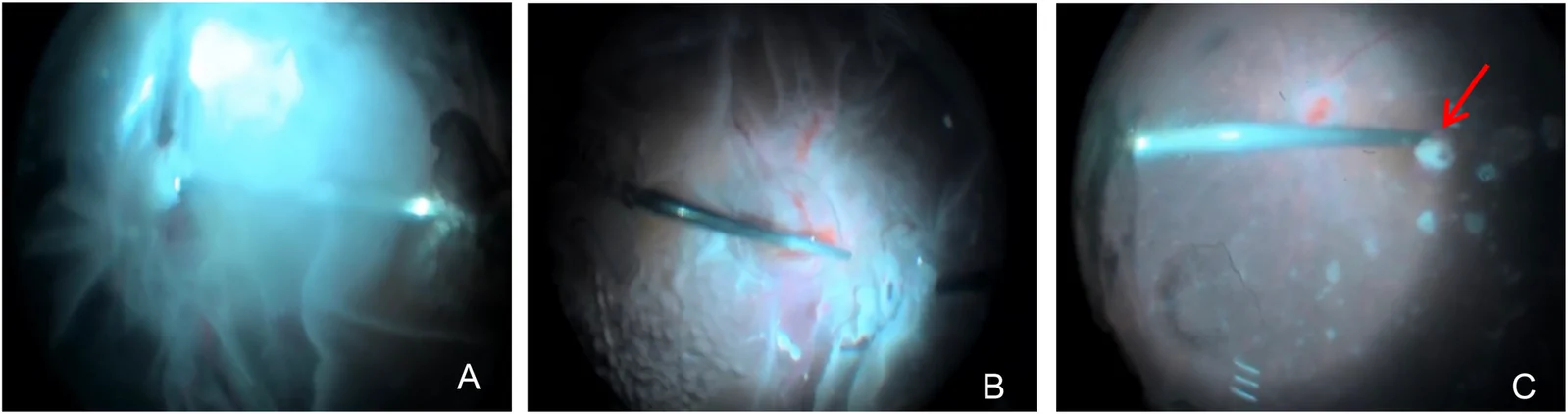
During the operation, dense mass like pus plugs were found in the vitreous cavities of both eyes, tightly adhering to the surface of the retina, forming a white membranous change **(A)** after cleaning, scattered retinal tears were observed in the right eye, and retinal detachment was observed throughout the circumference **(B)** A white mass like lesion appeared in the temporal side of the macular area of the left eye, growing from below the retina (red arrow), breaking through the retina and extending to the front of the retina.

**Figure 5:**
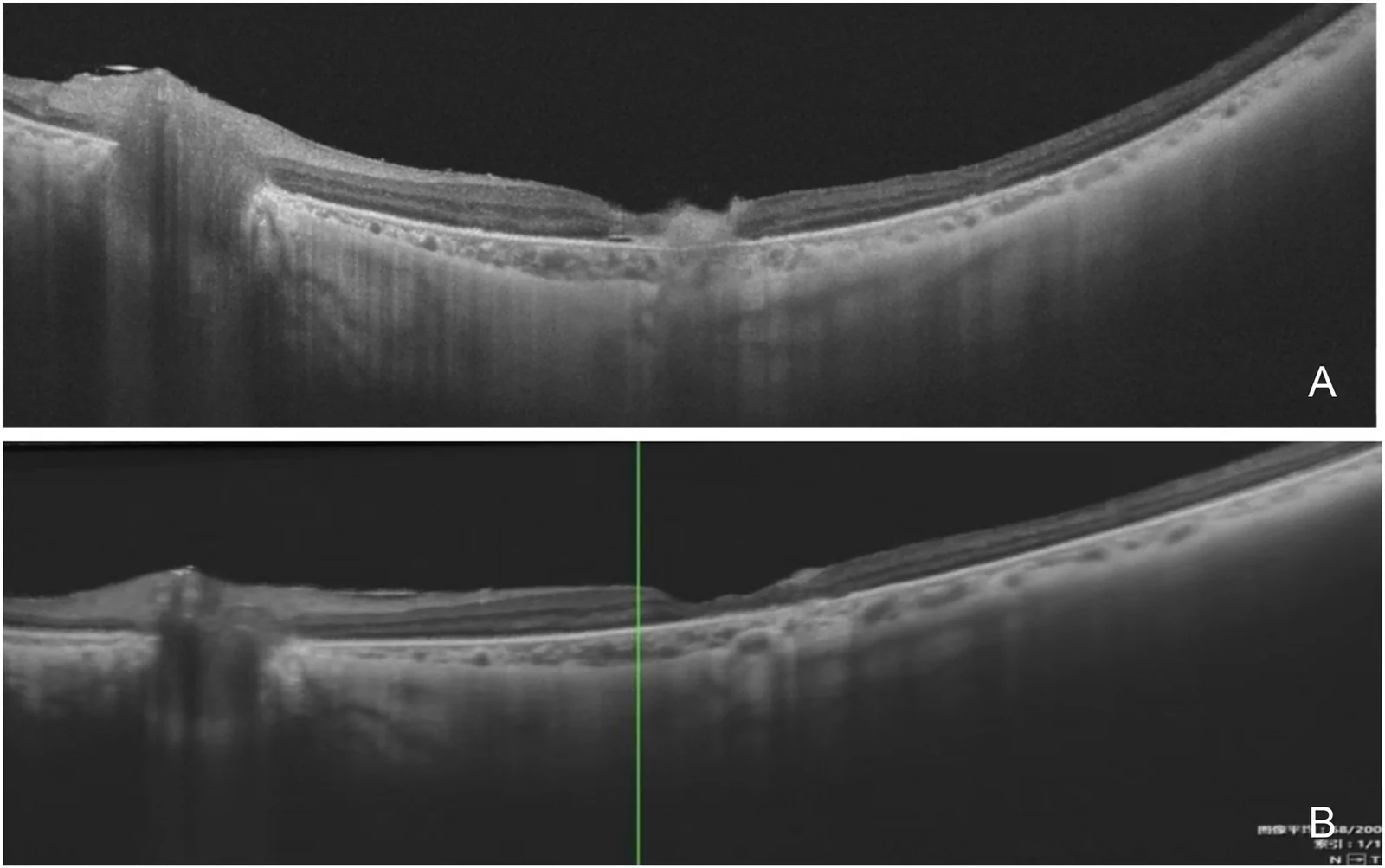
OCT in the first week after left eye surgery shows a dense block like high reflection signal in the macula retina **(A)**; three months after left eye surgery, OCT showed that the clustered lesion in the macular area was significantly absorbed compared to before **(B).**

## 3 Discussion

EFE is a vision-threatening infectious ocular disorder. Major risk factors for its development include diabetes mellitus, renal failure, long-term use of corticosteroids or immunosuppressive agents, prolonged intravenous catheterization after major surgery, urinary tract catheterization, and intravenous drug abuse (6). This patient is a 72 year old male with a history of diabetes. Before the onset of the disease, he had undergone surgery for kidney stones and kidney abscesses. After the surgery, he developed recurrent fever and low back pain, suggesting that there may be fungal infection in the urinary system and blood dissemination. This is consistent with the report of endogenous fungal endophthalmitis after the surgery for urinary stones (7–10). This case had an early onset of unclear vision in the right eye, manifested as signs of anterior uveitis. The initial diagnosis was “right eye uveitis” and local application of glucocorticoids. According to literature reports, EFE is easily misdiagnosed as uveitis in the early stage, with a misdiagnosis rate of 50% to 93.3% (11, 12). This case was diagnosed after traveling to multiple hospitals for more than 4 months, confirming this clinical dilemma.

When ocular inflammation of unclear etiology occurs and fails to respond to conventional anti-inflammatory and antiviral therapy, fungal endophthalmitis should be strongly suspected, and intraocular fluid testing should be performed promptly. mNGS as a new generation pathogen detection technology, has shown significant advantages in intraocular infections. A recent study involving 31 cases of fungal endophthalmitis showed that the mNGS positivity rate was 90.3% (28/31), significantly better than traditional culture (9.1%), and the detection turnover time could be shortened to less than 24 h. The positivity rate of mNGS in endogenous fungal endophthalmitis can reach 100% (13). However, mNGS still has certain limitations, as it can only detect nucleic acids and cannot distinguish between colonization, contamination, and active infections. Its detection efficiency for pathogens such as fungi is limited, and it lacks the ability to detect conventional drug resistance. In this case, mNGS used a sequencing platform (Nextseq 550) and a negative control (NTC) throughout the entire detection process. The reporting threshold was set as follows: the target microorganism was not detected in NTC and ranked in the top 10 for genome coverage, or the RPM sample/RPM NTC ratio was greater than 10.In this case, *Candida genus* (sequence number 3) and *Candida tropicalis* (sequence number 1) were detected in the vitreous humor mNGS, and their detection in sterile body fluid should be highly suspected as pathogenic pathogens. But the fungal culture results of the vitreous humor sent for testing at the same time were negative. Moreover, the patient responded well to the combination of voriconazole and vitrectomy, which to some extent confirms the clinical diagnosis of EFE. At the same time, cytokine testing showed that IL-6 (4,962.94 pg/mL) and IL-8 (1,854.56 pg/mL) were extremely elevated, IL-10 was mildly elevated (26.37 pg/mL), and the IL-10/IL-6 ratio was only about 0.005. Research has shown that significantly elevated levels of IL-6 and IL-8 are important biomarkers for infectious endophthalmitis, and a low IL-10/IL-6 ratio can effectively exclude primary vitreoretinal lymphoma (usually > 1.0) (4, 5), which is consistent with mNGS results.

In recent years, epidemiological data has shown that the overall incidence of ocular involvement in patients with *candidemia* is about 10.7%, with a strictly defined incidence of *candidal* endophthalmitis (chorioretinopathy with vitreous involvement or intravitreal abscess) of about 1.8%. It is worth noting that the incidence of endophthalmitis in Asia (3.6%) is significantly higher than in Europe and the Americas (1.4%) (14), which may be related to differences in the distribution of *Candida species* in different regions. A large retrospective study involving 674 patients with candidemia showed that infection with Candida albicans or Candida tropicalis was an independent risk factor for developing endophthalmitis (sub distribution hazard ratio 7.90, 95% CI: 2.42–25.75). The incidence of intraocular inflammation in patients with Candida albicans and Candida tropicalis is as high as 7% (16/245) and 10% (13/126), respectively, while Candida albicans, Candida krusei, and Candida parapsilosis (0% -3%) rarely cause intraocular inflammation (15). This discovery suggests that the dual challenges of high drug resistance rate (>30%) and high risk of ocular involvement of tropical Candida in China make it a pathogen that requires special vigilance in clinical practice (11).

The most noteworthy clinical feature of this case is the detection of *Candida albicans* (++) in renal and lumbar lesion cultures, while tropical *Candida albicans* was detected in intraocular vitreous mNGS. Multiple possibilities need to be analyzed for this phenomenon. (1) Mixed bacteremia with tissue tropism differences: The patient has hematogenous dissemination of both *Candida albicans* and *Candida tropicalis*, and different strains selectively colonize different organs due to tissue tropism differences. Literature reports that *Candida albicans* is the main pathogen of endogenous candidal endophthalmitis, accounting for over 90%. Although hematogenous endophthalmitis caused by *Candida tropicalis* is rare, it has been reported in literature. Cohen and Montgomerie reported two cases of *Candida tropicalis* hematogenous endophthalmitis and reviewed 12 cases in the literature, whose clinical manifestations were similar to those of *Candida albicans* endophthalmitis (16). There are also literature reports that mixed infections of *Candida albicans* and *Candida tropicalis* can occur in invasive fungal diseases such as endocarditis (17). At present, the evidence is insufficient to confirm the mechanism of tissue tropism, and further research is needed to verify it.(2)Sequential infection: The patient first develops candidemia and colonizes the kidneys and lumbar spine, and later, due to new invasive procedures or changes in immune status, tropical Candida enters the bloodstream and spreads into the eyes. (3) Multiple focal infections of independent origin: Candida infections in different parts of the body originate from different pathways (such as urinary tract derived Candida albicans and exogenous tropical Candida), and tropical Candida in mixed infections. (4) Related factors for detection: Only culture method was used for lumbar lesions, without mNGS detection, which may have missed the detection of tropical Candida in mixed infections; The detection of tropical Candida in intraocular fluid mNGS and negative fungal culture may be due to low pathogen load or insufficient strain vitality.

Based on the course of the disease in this case - systemic infection (fever, lower back pain) occurred more than 4 months before eye symptoms, renal and lumbar lesions were chronic enveloped infections, and the eyes were acute severe infections - Mixed bacteremia accompanied by differences in bacterial tissue tropism can serve as a reasonable speculative explanation. However, it should be clearly pointed out that this inference still lacks conclusive microbiological evidence: due to the lack of synchronous mNGS testing for each lesion, missing blood fungal culture results, and lack of fungal culture validation for intraocular fluid, other possibilities such as contamination, sequential infection, or independent origin cannot be completely ruled out at present. In the future, it is recommended to perform mNGS and fungal culture simultaneously on multiple specimens to improve the detection rate of mixed infections.

In response to the complex mixed infection of chronic Candida albicans deep infection combined with acute tropical Candida endophthalmitis in this case, a treatment plan was developed in collaboration with pharmacists and infectious disease doctors from our hospital's pharmacy department. Ultimately, the use of a combination therapy of caspofungin and voriconazole was established, which has clear pharmacological and evidence-based rationality. According to the 2016 IDSA guidelines, caspofungin is considered a first-line drug for echinocandins (18). The 2021 Consensus on Invasive *Candidiasis* in China states that voriconazole has excellent blood eye barrier penetration and is the preferred systemic medication for candidal endophthalmitis. It can compensate for the insufficient concentration of caspofungin intraocular drugs and can be combined with intravitreal injection of amphotericin B (5–10 *μ* g/0.1 mL) or voriconazole (100 μ g/0.1 mL). For lesions with obvious vitreous involvement or threat to the macula, intravitreal injection is an important adjuvant therapy (19). However, it should be frankly pointed out that there is currently a lack of large-scale randomized controlled trials to support the use of voriconazole combined with caspofungin for intraocular inflammation caused by mixed infections of *Candida albicans* and *Candida tropicalis.* The synergistic effect of the combination of two drugs is mainly based on *in vitro* pharmacological inference. The use of a combined approach in this case is based on individualized treatment decisions based on the complexity and difficulty of the condition.

Systemic antifungal treatment should ensure a full course of 8–12 weeks (20). Timely vitrectomy is the key to saving visual function, as it can directly remove infected lesions and cloudy vitreous bodies. Vitrectomy is of great value for cases of extensive vitreous inflammation, retinal detachment, or ineffective drug treatment (11). After the surgery, the left eye visual acuity in this case recovered from manual/anterior vision to 0.2. During the recent follow-up up to 8 months after the surgery, the left eye visual acuity further improved to 0.25, and the patient's condition remained stable with no signs of infection; Although the right eye still has no light sensation, the infection has been controlled, which verifies the effectiveness of comprehensive treatment.

The limitations of this case include: (1) single case report with limited extrapolation of conclusions; (2) The intraocular fluid only underwent mNGS detection, and the fungal culture result was negative, lacking cross validation of the culture method; (3) Lumbar lesions were only cultured without synchronous mNGS detection; (4) The missing blood culture results from each period cannot confirm the existence of mixed bacteremia; (5) Lack of fungal drug sensitivity test results; (6) The follow-up time is still short (8 months after surgery), and the long-term recurrence risk needs further observation.

This case report was approved by the Clinical Research Ethics Committee of the Hebei Chest Hospital. Compliant with the ethical requirements of the 《Helsinki Declaration》.

## 4 Conclusion

EFE has a insidious onset and a high misdiagnosis rate. For high-risk individuals with unexplained eye inflammation, pathogen testing should be completed as soon as possible. When the systemic and intraocular pathogens are inconsistent, it is necessary to carefully analyze various possibilities such as mixed infection, sequential infection, and independent origin. MNGS is a powerful tool for rapid pathogen identification, but the results need to be combined with clinical comprehensive judgment. Even if the fungal culture is negative, the possibility of EFE cannot be completely ruled out. The comprehensive plan of using systemic combined local antifungal drugs + vitrectomy, foot therapy, and standardized follow-up is the key to controlling inflammation and saving visual function. Clinical physicians need to establish a global mindset and pay attention to the intrinsic relationship between systemic lesions and eye damage.

## Data Availability

The original contributions presented in the study are included in the article/Supplementary Material, further inquiries can be directed to the corresponding authors.
